# Facilitating equitable community-level access to maternal health services: exploring the experiences of Rwanda’s community health workers

**DOI:** 10.1186/s12939-019-1065-4

**Published:** 2019-11-26

**Authors:** Germaine Tuyisenge, Valorie A. Crooks, Nicole S. Berry

**Affiliations:** 1 Department of Geography, Simon Fraser University, 8888 University Dr, Burnaby, BC V5A 1S6 Canada; 2 Faculty of Health Sciences, Simon Fraser University, Burnaby, Canada

**Keywords:** Maternal community health workers, Maternal health services, Equity, Middle and law income countries, Rwanda

## Abstract

**Background:**

In Rwanda, community health workers (CHWs) are an integral part of the health system. For maternal health, CHWs are involved in linking members of the communities in which they live to the formal health care system to address preventative, routine, and acute maternal care needs. Drawing on the findings from in-depth interviews with maternal health CHWs and observational insights in ten Rwandan districts, we identify specific strategies CHWs employ to provide equitable maternal care while operating in a low resource setting.

**Methods:**

Using case study methodology approach, we conducted interviews with 22 maternal health CHWs to understand the nature of their roles in facilitating equitable access to maternal care in Rwanda at the community level. Interviews were conducted in five Rwandan districts. Participants shared their experiences of and perceptions on promoting equitable access to maternal health service in their communities.

**Results:**

Four key themes emerged during the analytic process that characterize the contexts and strategic ways in which maternal health CHWs facilitate equitable access to maternal care in an environment of resource scarcity. They are: 1) community building; 2) physical landscapes, which serve as barriers or facilitators both to women’s care access and CHWs’ equitable service provision; 3) the post-crisis socio-political environment in Rwanda, which highlights resilience and the need to promote maternal health subsequent to the genocide of 1994; and, 4) the strategies used by CHWs to circumvent the constraints of a resource-poor setting and provide equitable maternal health services at the community level.

**Conclusion:**

Rwanda’s maternal CHWs are heavily responsible for promoting equitable access to maternal health services. Consequently, they may be required to use their own resources for their practice, which could jeopardize their own socio-economic welfare and capacity to meet the demands of their families. Considering the unpaid and untrained nature of this position, we highlight the factors that threaten the sustainability of CHWs’ role to facilitate equitable access to maternal care. These threats introduce turbulence into what is a relatively successful community-level health care initiative.

## Background

Maternal health remains one of the most pressing global health issues [1], especially in low and middle income countries where 99% of maternal deaths occur globally [2]. Most maternal deaths are preventable, provided that adequate and timely access to skilled care is available [3–5]. Rwanda has made efforts to increase women’s access to skilled birth attendants, and its maternal mortality ratio decreased dramatically from 1020 in 2000 to 290 in 2015 [2]. Binagwaho et al. note that incorporating informal (i.e., not formally trained or paid) community health workers (CHWs) into the health system was one of the strategies that supported, and is continuing to support, this significant decrease [6].

In Rwanda, maternal health CHWs (MH-CHWs), commonly referred to as *Animatrice de Santé Maternelle* are volunteers involved in linking members of communities in which they live to the formal health care system to address preventative, routine, and acute maternal care needs [7]. Similar to other low and middle income countries where CHWs are an integral component of health systems, Rwanda’s MH-CHWs are also typically the first point of contact with the health system for most women seeking maternal health care [8–10]. As volunteers elected within their own communities to promote equitable access to maternal healthcare, MH-CHWs play a vital role in ensuring that such services are reasonably accessible to the community members they serve [11, 12].

Globally, CHWs have been recognized as key players in health promotion programs, particularly in low and middle income countries [13]. Their role supports the development of community-based primary health care that was laid out initially in the World Health Organization’s Declaration of Alma Ata [14]. CHWs’ roles and responsibilities vary by country and by community [15]. CHWs have helped to increase equity in access to health care at the intra-national level throughout the Global South, especially in locations that are remote or have health human resources shortages [16]. The World Health Organization recommends that CHW based maternal health interventions should include: education and promotion of reproductive health; promotion of maternal health care-seeking behaviors; and passive support during the delivery and administration of misoprostol to prevent postpartum hemorrhage [17]. These practices emphasize the importance of CHWs’ familiarity with maternal health in the context of communities they serve as well as the importance of promoting health-seeking behaviors and strategies for enhancing equitable access. In effect, these practices form the basis of MH-CHWs’ roles in Rwanda, as community-level providers of maternal care.

The involvement of CHWs in different countries’ health systems was given great attention in the Millennium Development Goals era [18]. During that period (1990–2015), an agenda was advanced to make progress on several health-related goals, including millennium development goal 5 (improve maternal health). A number of countries have greatly benefited from the services provided by CHWs and made progress in terms of enabling access to skilled birth attendance and overall maternal health outcomes and thus achieving goal 5 [19, 20]. The Lady Health Workers Program in Pakistan (LHWP) is one of the most successful CHW programs in maternal health. It aims to provide maternal health services to poor and underserviced communities of the country and focuses on family planning, antenatal care and the use of skilled birth attendants [21]. Studies have shown that maternal mortality rates decreased in areas covered by the LWHP (60% of the country) as compared to areas not covered [21]. Even though this reduction in maternal mortality rates cannot be attributed to LWHP program solely, it is important to acknowledge that the services they provide – including providing access to modern reversible contraceptives, attending deliveries, maternal health education, tetanus toxoid coverage - are known to be important contributors to reducing maternal mortality [22]. Rwanda is another country that saw improvement in maternal health during the Millennium Development Goals’ era, much of which was purportedly driven by the rise of the country’s CHW program and the recruitment of MH-CHWs throughout rural and urban areas [7].

Rwanda’s historical, geographical, socio-demographic and economic contexts make it a distinctive case study in terms of understanding MH-CHWs’ involvement in facilitating maternal health and enabling equitable access to the health system. Rwanda is a small, mountainous, landlocked country that has a population of about 12 million [23]. The country’s rebuilding subsequent to the 1994 Genocide called for community collaboration in different sectors, including health [6]. By 2013, more than a decade after this rebuilding effort started, its doctor-patient ratio was only 1 per 16,046 people and its midwife-patient ratio was 1 per 18,790 people, while there was a ratio of 1 nurse per 1227 people [6]. Continuing health worker shortages have only increased the need to implement a CHW model throughout the country, and to promote the use of MH-CHWs as a way of improving equitable access to maternal care [7].

Rwanda’s MH-CHWs are in charge of maternal health promotion and provide monthly reports on selected health issues to community health centres. Each health centre oversees 120–200 CHWs, including 35–50 MH-CHWs, operating in its catchment area. Health centres report their activities to district hospitals, which in return report to the Ministry of Health. At the community level, there is one MH-CHW in each village about 300–450 residents. Every MH-CHW is elected by the members of the village during community gatherings [13]. MH-CHWs’ specific roles involve educating women on the timely use of health facilities for maternal health services; providing women with pertinent information; recording women’s appointments and providing appointment reminders; and accompanying women to health centres for delivery. Further, MH-CHWs encourage men’s involvement in maternal health as well as overall family support as a way of decreasing avoidable barriers to care [24, 25]. There are two major ways MH-CHWs interact with women: during home visits and community gatherings. For home visits, MH-CHWs either visit each household in their catchment area at least once a month or they receive women who need maternal health services in their own homes [26]. At community gatherings, MH- CHWs provide village members with relevant maternal health information [7, 26].

Since its implementation into the health sector in 2005, the CHW program has contributed to: the increase in antenatal care; the increase in facility-based deliveries from 45 to 69% between 2005 and 2010; supporting the implementation of maternal death audits at the community level; the provision of family planning services at the community level, hence increasing the total rates of family planning usage [7]. Despite these significant achievements, the CHW program is a voluntary one and there is no compensation provided for being elected into this role. Early on in developing the CWH program the Ministry of Health had implemented a performance-based financing system for community health services [7]. The payments were to be provided to CHWs depending on the financial resources of each health centre [8]. When the performance-based financing system was first introduced, the idea was to provide funds through cooperatives whereby the CHWs of a health centre would form a cooperative and use the funds to implement income-generating projects [7]. Most cooperatives were not successful due to the irregularity in receiving funds, insufficient financial literacy among cooperative members, and poor accountability in performance and thus this funding initiative was not sustained [7, 8]. While acknowledging MH-CHWs’ remarkable contributions to the reduction of maternal mortality and promotion of overall maternal health in Rwanda, there is a need for research that documents *how* they actually facilitate equitable access to maternal care (i.e., how they help with eliminating or mitigating unnecessary or unfair social, financial, spatial, and other barriers to accessing maternal care [7, 27, 28]. Herein we explore this knowledge gap, considering how MH-CHWs’ facilitate such care while negotiating settings marked by resource scarcity. By ‘resource scarcity’ we to acknowledge that Rwanda’s MH-CHWs receive little-to-no material support (e.g., cell phones, medical equipment, clothing) to facilitate care, that they receive no financial remuneration for taking on this role, that they do not have formal health worker training, and that they voluntarily provide care within the context of an inequitable health and social care environment. There is also a pressing need to identify ways that MH-CHWs can continue to improve Rwanda’s maternal health indicators given that there is no indication this program will be changed or eliminated in the near future and that these indicators need to be strengthened. For example, the most recent demographic health survey [23] recorded a maternal mortality ratio of 290 per 100,000 live births, placing Rwanda among the countries with the highest maternal mortality globally. This ratio calls for both strengthening existing strategies and developing new programs in order to improve maternal health in the country and work towards achieving the Sustainable Development Goal of reducing the global maternal mortality ratio to less than 70 per 100,000 live births by 2030 [29].

This analysis supports this goal of improving Rwanda’s maternal health outcomes by considering maternal health promotion as an important determinant of health and exploring the needs of women and the resources available to them in order to ensure that they receive adequate care. Studies in other low and middle income countries show that community health workers provide maternal health services by either visiting households in their catchment areas or by having community members come to the homes of community health workers for health services [21, 30]. In addition, mobile technology [31, 32] and community gatherings [33, 34] are used as ways of providing maternal health education and the reporting of maternal health indicators. There is still a gap in understanding the effectiveness of such facilitating factors in environments of resource scarcity considering that CHWs are unpaid and untrained. The findings of this qualitative analysis may have transferability to other contexts where CHW programs have been implemented and can thus assist with addressing these wide knowledge gaps.

## Methods

The goal of this exploratory qualitative case study was to understand how MH-CHWs facilitate equitable access to maternal care in Rwanda at the community level within a context of resource scarcity. The case study methodology lends itself to the study of complex aspects of social or human problems in various contexts [35]. This approach is appropriate when seeking to answer *how* and *why* questions [36], which is consistent with our study. Case studies often use multiple methods in order understand the full context of the issue under study, and in the current study this involved conducting semi-structured interviews with MH-CHWs in their local villages; holding informal conversations with policy officials and health system administrators; and visiting villages to tour hospitals and community health centres with local hosts. In addition, we reviewed appropriate maternal care policy and practice guidelines.

### Study setting

This study was conducted in three provinces of Rwanda: Northern Province; Southern Province; and Kigali Province. We purposefully selected these three provinces for inclusion to capture geographical and socio-demographic differences that characterize Rwanda’s diversity. Data collection took place in particular districts within these provinces (see Table 1), with input and approval from national and district health officials: Gakenke and Rulindo districts (Northern Province); Nyarugenge and Gasabo (Kigali); and Ruhango (Southern Province).

**Table 1:** Districts where data were collected

District	Land size (Km^2^)	Population size [37]	Population Density [37]	# of district hospitals	#of M-CHWs	# of health centres	# of visits per district
Nyarugenge	134	284,561	2125	2	350	10	5
Gasabo	430.30	530,907	1237	2	486	16	5
Gakenke	704.06	338,586	481	2	617	23	6
Rulindo	567	287,681	507	2	494	21	6
Ruhango	626.8	318,885	510	2	533	15	6

### Participant recruitment

Recruitment was conducted by CHW coordinators working in local health centres in the districts of focus. These coordinators were first contacted by on-site representatives of a large, international research and intervention program designed to improve maternal health (to which this study contributes). The representatives informed the coordinators about the study as well as how to make arrangements for MH-CHWs to participate. We explained that we wished to interview any MH-CHWs who expressed an interest in participating in a face-to-face interview during our six-week on-site data collection period. Thus, data collection was driven by a temporal cut-off rather than reaching a target number of participants. CHW coordinators distributed written and verbal information about the study to MH-CHWs, and those who were interested in participating in the study informed the coordinator. To build trust with potential participants, the interviewer – who is the lead author - arranged to meet with interested MH-CHWs to explain the study further, answer any questions, and schedule interviews at a convenient time. In order to maintain confidentiality, the CHW coordinators and health centres were never informed who ultimately agreed to participate.

### Data collection

In-depth interviews were conducted by the first author, who is a Rwandan national, between July and August, 2017 with MH-CHWs in the five districts of focus. Participants chose the interview location and, to minimize the burden on their time, most MH-CHWs chose to be interviewed at the nearest health centre on a day when they had already planned to accompany a pregnant woman. Some interviews took place at a participant’s house. Interviews were conducted in the Kinyarwanda language (the official national language taught in all schools) and began with a review of the study’s goals and a discussion of participants’ rights in the study. The interviewer then obtained participants’ verbal consent for participation. Basic demographic information was collected at the start of the interview (e.g., age, marital status, number of children, education attainment, profession, and years of experience as a MH-CHW) to better understand information and gather the context of the interview. Next, open-ended questions were asked to elicit experiential insights from MH-CHWs, including their understanding of maternal health and the factors that contributed to the use of, access to, and delivery of maternal care services.

Interviews were first conducted in Gasabo district, Kigali, to pilot the recruitment process as well as the guide. Following completion of these initial interviews, and with the input of other research team members, the semi-structured guide was revised in order to refine the questions asked. Additional questions were added that explored how and why women became MH-CHWs as this issue was emerging as both complex and significant. Interviews lasted between 45 and 70 min and were audio-recorded. Detailed field notes were kept throughout the data collection period, with entries made following each interview regarding body language and conversational tone, among other factors, and following site visits to health care centres.

A total of 22 interviews were conducted with MH-CHWs. All were women between the ages of 33 and 52. Most participants reported farming as their main income-generating activity. The remaining few reported being tradespeople, artisans, or involved in some other form of waged employment. They each had between two and eight children. Their education ranged from the completion of primary school to a few years of secondary school. None were formally trained health workers or held health worker certifications. The time they had been volunteering as MH-CHWs ranged from as little as 3 months to 8 years at the time of the interview, with the average being just under 3 years.

### Data analysis

Data were analyzed using thematic analysis, a technique that involves identifying patterns in datasets that characterize aspects of the phenomenon being explored and comparing those patterns to the findings of existing studies in order to understand how they relate to existing knowledge [38]. The interviewer simultaneously transcribed and translated the audio recordings into English after completion. The transcripts were stored and organized with NVIVO™ software, which is a qualitative data analysis program. The process also included uploading field notes into the same data management platform. Following data preparation, team members independently reviewed selected transcripts to identify emerging issues in the dataset to be explored through analysis as well as determine dominant themes around which to organize key findings. Next, team members met face-to-face to identify commonalities emerging from these independent reviews to confirm the scope and scale of dominant themes, which is an important part of thematic analysis.

The insights that had been generated through independent review were shared in a team meeting and used to develop a coding scheme that incorporated inductive and deductive codes for thematic analysis. Coded data extracts were next reviewed by team members to determine sub-themes and the relationships between different themes for data interpretation. It was through this process that a focus on community context and the strategic ways in which MH-CHWs facilitate equitable care access in an environment marked by scarcity emerged, which we explore herein.

### Ethical considerations

Ethical approvals to conduct this study were obtained from the Research Ethics Boards of Simon Fraser University and the Ministry of Education of Rwanda. MH-CHWs were assured that their participation was voluntary and confidential, and that they were free to withdraw from the study at any time. In order to acknowledge their contribution to the research, act consistent with cultural protocols and in light of participants’ socio-economic positions, a food item was given to each participant to acknowledge the value of their time and importance of their contribution to knowledge (e.g., a bag of rice or sugar).

## Findings

In this section, we explore four key themes that emerged during the analytic process that characterizes the contexts and strategic ways in which Rwandan MH-CHWs facilitate equitable access to maternal care in an environment of scarcity and in the context of barriers identified by the participants to affect women’s access to care. These themes are: 1) community building, or specifically the role of community participation in promoting maternal health and facilitating MH-CHWs’ roles; 2) physical landscapes, which serve as barriers or facilitators both to women’s care access and MH-CHWs’ equitable service provision; 3) the post-crisis socio-political environment in Rwanda, which highlights resilience and the need to promote maternal health subsequent to the genocide of 1994; and, 4) the strategies used by MH-CHWs to circumvent the constraints of a resource-poor setting and provide equitable maternal health services at the community level. These themes are underpinned by existing literature on MH-CHWs’ roles and experiences of service provision in low and middle-income countries, which we explore in the discussion. Throughout the remainder of this section we include direct quotes to highlight participants’ voices regarding the issues being explored.

### Community building

Participants strongly believed that advancing equitable access to maternal health in Rwanda required the involvement of the entire local community. This was best achieved when initiatives were implemented to promote maternal health while strengthening community relationships. For example, one MH-CHW described her involvement in a healthy cooking program that illustrated such a holistic initiative: *“In our village, we do monthly cooking demonstrations. Every woman contributes 100 francs [USD$0.03] at each gathering and brings other food items … we teach them how to make healthy meals … This has helped to enhance our role in the community.”* Participants believed that in their capacities as MH-CHWs, they were responsible for building community networks and collective initiatives that contributed to achieving maternal health promotion objectives. For example, it was regularly explained that one of the greatest challenges pregnant Rwandan women faced was the cost of purchasing health care. Women were encouraged to have health insurance, and for most families who could afford to pay for premiums up-front, being part of community associations helped them to save and pay for health insurance in a timely fashion. One participant stated that women in her village had an association that *“gathers once a week … each woman gives weekly contributions and we use those savings to pay for health insurance … this is helpful especially for bigger families who cannot afford to pay the premiums all at once.”* By supporting such initiatives, MH-CHWs were facilitating their own roles in supporting equitable access by ensuring that all women were able to gain access to formal maternal care services at health centres and hospitals.

In order to strengthen community health programs, local village leaders provided support to MH-CHWs that enabled maternal health efforts to be integrated into other community promotion programs. Local leaders, from village chiefs to community security officers at the village level were familiar with the day-to-day lives of their community members and collaborated with MH-CHWs on some maternal health-related issues to make sure all women in the village received the care they need. This was emphasized by one participant: *“We work with local authorities... When they notice problems in a family they inform us.”* Participants highlighted how local leaders updated them about demographic changes in their communities: *“Sometime new people move in … the village chief helps me to get information about these families; he calls me and presents me their situations and I arrange to go visit them.”* Moreover, all participants discussed calling for local authorities’ help in cases where they needed trusted support. For example, “*When a woman refuses to attend antenatal care, I report it to the village chief... I tell him/her because she [the woman] may die if she doesn’t consult the health centre or by trying to abort... together we go talk to her.”* Most participants stressed how collaboration with community leaders established a strong foundation for equitable primary health care at the community level. Collaboration was central to identifying people in need of care, enabling MH-CHWs to fulfil their own voluntary role and carry out their responsibilities to promote maternal health.

### Physical landscapes

Rwanda’s mountainous landscape played an important role in shaping women’s clinical utilization and also MH-CHWs’ service delivery. One of the MH-CHWs’ roles was to encourage women to use health services for prenatal, delivery and post-natal care. However, most participants acknowledged Rwanda’s challenging, highly mountainous landscape as impacting women’s attendance at health services, indicating that spatial access sometimes limited the use of such services. As mentioned by most participants, there were some remote communities where health centres were located a considerable distance away from women’s homes, making it a difficult for both women to visit them and difficult for MH-CHWs to visit pregnant women. A participant stressed that it can take: “*between 40 and 45 minutes for a strong person to get to the health centre. For physically weak people, it takes them about an hour because they have to walk up to the top of the hill.*”

Participants had developed a number of non-resource-intensive strategies to facilitate equitable maternal care access to assist women with overcoming spatial access challenges. For example, most participants accompanied women on walks to clinic appointments to support them on the journey and assist with navigating difficult pathways. Wherever possible, individual or taxi bicycles were used to get women to health centres – sometimes paid for out-of-pocket by MH-CHWs. However, participants also indicated that bicycles were unable to navigate particularly challenging terrain: “*the road is very ascending and even a bicycle cannot take a patient up there due to the long distance*.” In addition, many participants talked about how weather compounded the maternal care access challenges brought about by Rwanda’s physical landscape. For example, participants mentioned that some roads can become slippery or damaged during rainy periods, making it harder for women to get to health facilities. Thus, some participants talked about being attentive to weather reports in order to plan the best times or routes to visit women or assist them with getting to health centres.

In some cases, participants drew on their personal resources to overcome the spatial barriers to accessing maternal health care that may have negatively impacted their abilities to support care provision at the community level. Journeys to get women to health centres could be long and are sometimes undertaken in the dark. A few participants talked about arranging for their own family members to accompany them when walking long distances, especially in the dark. Some participants also reported bringing personal flashlights to enhance safety and assist with navigating challenging pathways. In some cases, MH-CHWs drew on their personal finances in order to facilitate access across challenging terrain in difficult weather conditions: “*When the weather is bad I may hire a motorcycle to take a woman to the health centre, knowing that she doesn’t have money to pay for transportation and I pay … she may or may not reimburse me.”* As noted in this quote, there was typically no expectation of reimbursement and many participants understood that their commitment to improving maternal health would require them to draw on their own personal resources from time-to-time.

### Post-crisis social-political environment

Bringing communities together to ensure the sustainability of health system was among the development programs that became imperative to rebuilding Rwanda after the genocide of 1994. Participants acknowledged their roles and responsibilities in bringing communities together to promote maternal health and facilitate equitable access to care in their villages. Several participants stressed that promoting community unity was one of the assignments they received as MH-CHWs: “*We receive training on maternal health … during each training they emphasize on our role as the eyes of our communities … Our role is to make sure that everyone feels included in each program that we implement.*” Serving as the ‘eyes of the community’ helped MH-CHWs facilitate equitable access to maternal care at the community-level because doing so allowed participants to identify emerging local problems and priorities and assess their impact on the uptake of maternal care.

Most participants highlighted that the community gatherings championed in Rwanda’s era of unity functioned as ongoing channels through which they could provide maternal health messages. For example, one participant said that: “*After our monthly [community] cleaning session, there is a time dedicated to health education … we mention about maternal health because we know such a message will get to many people.”* Participants explained that bringing the community together for formal and informal discussion helped them to identify solutions for emerging issues, with maternal health being among the top priorities. MH-CHWs also held themselves accountable for the responsibility bestowed upon them by their communities: “*It is my responsibility to make sure that women have better health … if the community has elected me, I cannot give up even though it is a demanding job … it is my role to promote other women and my country.*” This sense of responsibility drove many participants to want to improve maternal care access and facilitate improved care access for their fellow community members.

### Circumventing constraints

Participants identified many strategies they used to deliver maternal care despite the resource scarcity that frequently characterizes the contexts in which they were situated. This context led many MH-CHWs to find ways to circumvent barriers to care delivery in order to facilitate equitable access to maternal care at the community level. For example, participants stressed the importance of acknowledging that taking on the MH-CHW role would likely involve some degree of loss of personal income in addition to the use of their own personal resources to facilitate care provision. As one participant explained: “*I always lose money when I am performing my duties as a MH-CHW … for example, even the time I have spent with you, I could have made lots of progress tailoring clothes … but I do not think like that … otherwise I would quit.*” For her, the benefit of MH-CHWs’ informal labour to the community was far more important than her personal income. This type of reasoning was made even more evident in cases where MH-CHWs used their own financial resources to facilitate care provision to circumvent the lack of resources they were provided with. As noted previously, most participants revealed that they had encountered situations where they had to use their own money to purchase things such as transportation, phone credits, and baby items or food for women they were serving who had even less financial means.

Most participants acknowledged that support from their own families was critical to the role they played in facilitating equitable maternal health service delivery in their communities. This was especially so given the unpredictable nature of their work and the scarcity of resources that characterized the context. “*You cannot perform well a MH-CHW without the support of your family members … you have to inform your husband of your moves and if he did not support you, he would not allow you to go.”* In a similar context, another participant said: “*Even your children have to agree because they help you with house tasks. My daughter used to help me during some CHW duties before she got married.”* All participants explained that they had to rely on their own families for support in order to provide maternal care to other women in the community. Some got family members to help at home with household and farming chores while they were away providing care, whereas others had spouses and other family members accompany them in MH-CHW-related activities.

## Discussion

The findings of this study reveal many of the complex ways in which Rwandan MH-CWHs facilitate equitable access to maternal health services at the community level. Broadly speaking, they accomplish this by assisting women and other community members with mitigating or eliminating avoidable barriers to access maternal health services MH-CHWs’ roles include overseeing different community initiatives that help to strengthen community relationships [39, 40]. The findings show that engaging in these wider activities builds trust with community members and helps MH-CHWs facilitate equitable access to care that local women need. MH-CHWs also promote community-building by working with other local actors, including other elected leaders, to encourage uptake of maternal care services and to identify women at risk of never accessing care. Other research has established that these types of roles are fundamental to promoting maternal health at the community level in a young, low income country with high fertility rates, such as Rwanda [40, 41]. Uniquely, the current analysis shows that MH-CHWs sometimes draw on their own personal resources in order to circumvent the barriers to care created by factors such as weather, getting to distant clinics, and caring for women with very limited financial resources. For example, they not only subsidize access to care through the use of their own wealth (e.g., time, money, cell phones, flashlights) but also draw on personal or family support networks to fulfill the demands of their voluntary labour. A strong desire to contribute to Rwanda’s rebuilding process during its post-crisis social-political environment and to support their local communities drive many women to take on the MH-CHW role in the first place, and thus the personal commitment to fulfilling this role demonstrated by drawing on one’s personal resources and networks may not be surprising.

Research elsewhere, including South Africa and India, has shown that CHWs’ consistent contact with communities is undertaken within a larger context of community members holding them responsible for improving access to maternal health services. Such expectations regarding the responsibilities associated with this role may compel CHWs to use their own readily-available personal resources to promote access to maternal services [34, 35]. The risk is that extending one’s personal resources and networks to facilitate such informal care provision may lead to burnout by CHWs or personal harms, such as debt. Though not examined in the current study, these are recognized outcomes of the physical, emotional, mental, and financial toll of providing unpaid care labour [42, 43], and thus the findings of the current analysis suggest that this may be an area worthy of policy exploration in Rwanda in order to avoid onset of caregiver burnout. In addition, the unpredictability of CHWs jobs and a lack of work schedules requires these informal caregivers to be available at any time of the day, which can jeopardize their paid job or family responsibilities [44]. The findings of the current analysis documented many specific examples. Identifying mechanisms to minimize the loss of personal income by Rwandan MH-CHWs is another area worthy of policy exploration or intervention.

Integrating the MH-CHW program into Rwanda’s health system was part of the country’s decentralization program, which aimed to increase the community’s participation in, ownership of, and accountability towards socio-economic development [45]. Rwandan MH-CHWs link communities and their members to the formal health system. This effectively positions them at frontline of promoting the benefits of maternal care and facilitating access to, and ultimately uptake of, such services [10, 25, 46]. Through community initiatives such as village meetings and cooking classes, the findings of this analysis show that MH-CHWs lead community gatherings as a means to promote healthy maternity practices in their communities. Such strategies have been proven to work in other contexts, such as Bangladesh [40] and Liberia [41]. These initiatives can help community members share experiences, learn about health resources available for community ownership to support the sustainability of the CHW program, and ultimately contribute to reducing maternal mortality. The findings of the current analysis show that by providing maternal health services in local communities, MH-CHWs also aim to promote equitable access to maternal health services through participating in, and leading, such community initiatives. In addition to the previous examples of using their own resources to subsidize poorer women’s care access, they also worked with other community leaders to identify marginalized women at risk of not using services, sometimes reaching out to them through community initiatives. In both of these examples, MH-CHWs use tailored strategies to improve equity of access to maternal care in their communities.

This analysis has raised important issues that can and should be explored through future research. Considering the deep individual responsibility that is built into Rwanda’s MH-CHWs program and the lack of structural or system-based resources available to those who provide such care, innovative low-cost initiatives that can support MH-CHWs’ service provision should be explored. Such research needs to be conducted from multiple perspectives in order to fully assess the potential costs and benefits of such initiatives. It is also essential to identify how sustainable financial, policy, and administrative support can be made available for such initiatives. In addition, to ensure that MH-CHWs’ roles are sustainable, actors within the health system should learn about what has and has not worked for various community health programs both locally and internationally. First, however, a stakeholder analysis would need to be undertaken to identify all those directly and indirectly involved in or impacted by the MH-CHW program in order to understand the scope of who would benefit the most from such learning. Thereafter, stakeholder’s voices should be sought out in research in order to inform striking the right balance between MH-CHWs’ community involvement and personal responsibilities. Such insight will assist with determining whether or not interventions can be implemented that support creating and maintaining such balance for Rwandan MH-CHWs and the informal care roles they balance against other life responsibilities. For example, these interventions could focus on identifying ways to enhance the training opportunities provided to MH-CHWs or to reward them through enabling skill building that is transferrable to paid employment contexts. Alternatively, MH-CHWs and their nuclear family members could be exempted from the modest annual fee that enables their own access to Rwanda’s formal care system, in recognition of their informal care work.

While the current analysis has explored the facilitation of equitable maternal care at the community level in Rwanda from MH-CHWs’ own perspectives, future research should explore how CHWs coordinators and others who provide formal maternal care services in Rwanda’s health care system view the effectiveness of MH-CHWs’ roles and responsibilities. Such insight will assist with identifying avenues for improvement, both for the work done by MH-CHWs and for improving equitable access, and also interventions to support MH-CHWs in their unpaid care work. We also propose that future research can examine the impact of MH-CHWs from the perspective of other community members in order to understand how these individuals understand MH-CHWs’ involvement in maternal health services. To this end, we suggest that research should be conducted with MH-CHWs families’, people who seek MH-CHWs services, and other community members – all of whom are members of MH-CHWs’ informal support networks. The findings of such research would reveal implications that the MH-CHW program has for different members of the community and potentially lead to identifying ways to strengthen the program and support the continued provision of informal care work.

## Conclusion

Rwanda’s MH-CHW program successfully promotes maternal health at the community level. However, as CHWs are untrained and unpaid, their roles are fragile and difficult to sustain. In low and middle income countries where health systems lack adequate resources, including human and financial resources, CHWs are heavily responsible for promoting equitable access to maternal health services and ensuring uptake of formal care. As a result, this analysis has shown that Rwanda’s MH-CHWs may feel compelled to use their own resources to facilitate care delivery, which could jeopardize their own socio-economic welfare and capacity to meet the demands of their families. To promote MH-CHWs’ roles and ensure that they are sustainable, policy revisions should enhance equity in MH-CHWs’ own responsibilities and support the roles they take on in and through community building. Stakeholders in maternal health, both within and beyond Rwanda, should consider the MH-CHW program as an example of a promising human resource that improves equitable access to maternal health and therefore needs to be strengthened. This will help facilitate progress in improving maternal health in low and middle income countries, where most of global maternal deaths are reported.

## Data Availability

The datasets generated and analyzed during the current study are not publicly available due to the respondents’ consent to use the data for this research specifically. Data can be available upon reasonable request to the corresponding author.

## Abbreviations

CHW: Community Health Worker
MH-CHWs: Maternal Health Community Health Workers
